# Two alternative chromatography methods assisted by the sulfonic acid moeity for the determination of furosine in milk

**DOI:** 10.1016/j.mex.2018.06.007

**Published:** 2018-06-15

**Authors:** Graciela Artavia, Lizeth Rojas-Bogantes, Fabio Granados-Chinchilla

**Affiliations:** a Centro Nacional de Ciencia y Tecnología de Alimentos (CITA), Universidad de Costa Rica, 11501-2060 Ciudad Universitaria Rodrigo Facio, San José, Costa Rica; b Centro de Investigación en Nutrición Animal (CINA), Universidad de Costa Rica, 11501-2060 Ciudad Universitaria Rodrigo Facio, San José, Costa Rica

**Keywords:** Furosine in powdered and fluid milk, Furosine, Milk, DAD/PDA detector, *p*-toluenesulfonic acid, Ion-pair liquid chromatography, Strong cation exchange chromatography, Adulteration

## Abstract

*N*^6^-(2-(2-Furanyl-2-oxoethyl))-l-lysine (furosine) is a deteriorative reaction product that is produced during heat treatment and storage of milk. This compound affects the quality of commercial dairy products. Accurate determination of furosine is necessary as it may serve as a measure of the degree of protein degradation in dairy products.

In this article, two HPLC based methods (1. a novel ion-pairing reagent 2. a strong cation exchange column) are proposed to quantify furosine. These methods were optimized and validated for their application to analyze fluid milk and dried milk powder.

• Two methods that can be used for routine milk quality control, including heat damage and adulteration, were developed.
• Compared to previous methods, the modified procedures herein using aromatic sulfonic acids (a pairing agent or covalently bound to a matrix on a strong cation exchange column) provide less expensive and more sensitive determinations.
• The identification and quantification of the furosine chromatographic signal was successfully achieved during analysis of commercial and spiked samples.

## Graphical abstract

**Figure:**
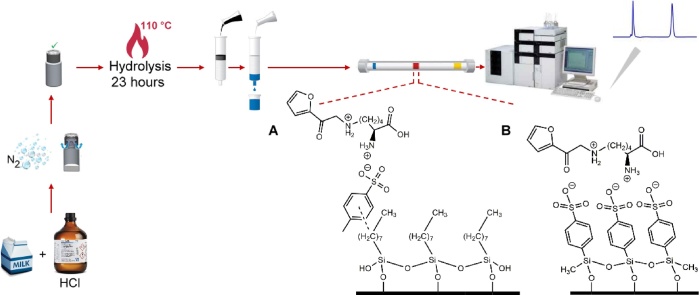

## Method details

### Background

Furosine is a compound formed in the early stages of the Maillard reaction (i.e., non-enzymatic browning reaction) [1,2]; it is formed after the hydrolysis of protein-bound lactosyl-lysine, commonly produced by heat exposure of milk. The lack of furosine in fresh milk means that its presence provides a marker indicating the application of heat treatment or prolonged storage [1,2]. Hence, it is classified as a Type II indicator (in the time-temperature integrators class) and thus is a suitable measurement of the nutritional quality and biological value of protein of a dairy product [1,3].

Miscellaneous methods for the determination of furosine in dairy include capillary zone electrophoresis [4] as well as a front-face fluorescence method [5], and recently, a stable isotope dilution assay coupled with tandem mass spectroscopy was reported for the simultaneous detection of several Maillard reaction products including furosine [6].

The most common methods for the determination of furosine are based on high- performance liquid chromatography (HPLC). For example, a previous report utilizing HPLC with an acetate buffer was applied to assay furosine in a variety of dairy products (and interestingly, dry dog food) [7]. In this regard, the ISO 18329 IDF 193 reference method [8] indicates the use of potassium chloride/acetic acid as furosine pairing agent. However, the technique fails to state, explicitly, which HPLC column was used for the separation. Few papers have used this approach [[9], [10], [11]], and other methods have been developed since.

Previously, sodium heptane sulfonate has been used as pairing agent by several research groups to aid the measurement of furosine in commercial milk [12], whipping cream [13] and retail whipping cream, coffee cream and condensed milk [14]. Ion exchange columns with post-column ninhydrin derivatizations have been used as an additional way to detect furosine in dried skimmed milk [15] and rumen undegraded protein [16]. Recently, an improved methodology was published (based on a modification from HPLC to UHPLC of an already established method [17]) to evaluate “heat load” in extended shelf milk samples, thus significantly reducing the analysis time during commercial milk assays [3].

Herein we report modifications of method ISO 18,329:2004(E). IDF: 193:2004(E). We substituted acetic acid and potassium chloride in the mobile phase for a solution of *p*-toluenesulfonic acid (TsOH) which interacts simultaneously both with the furosine and the C_8_ column. Alternatively, we used a strong cation exchange column (SCX, based on polymer bound sulfonic acid) for furosine determination. These modifications were optimized and validated, resulting in two reproducible and accurate approaches for the determination of furosine.

### Reagents

Furosine analytical standard was attained from Polypeptide Laboratories (hydrochloride, 99.4%, SC494, Strasbourg, France). Hydrochloric acid (HCl, ACS reagent, 37%), TsOH (402885, ACS reagent, ≥ 98.5%) and sodium 1-heptane sulfonate (H2766, HSA) were purchased from Sigma-Aldrich (St. Louis, MO, USA). HPLC grade acetonitrile (ACN, LiChrosolv^®^), and methanol (MeOH, ACS reagent) were acquired from Merck Millipore (Merck KGaA, Darmstadt, Germany). Ultra-High Pure Nitrogen was purchased from Praxair Technology Inc. (Danbury, Connecticut, USA). Ultrapure water [type I, 0.055 μS cm^−1^ at 25 °C, 5 μg L^−1^ TOC] was obtained using an A10 Milli-Q Advantage system and an Elix 35 system (Merck KGaA, Darmstadt Germany).

### Liquid chromatography equipment

A modular HPLC system (Shimadzu Prominence, Shimadzu Corporation, Kyoto, Kyoto Prefecture, Japan) equipped with a degasser (DGU-20A5), quaternary pump (LC-20AT), an autosampler (SIL-20 A HT), a system controller (CBM-20 A), a column oven (CTO-20 A), a photodiode array detector (SPD-M20AV) was used during all analysis. Chromatographic data management was performed using LC Solutions (Version. 5.2).

### Chromatographic conditions using ion pairing

**Fig. 1:**
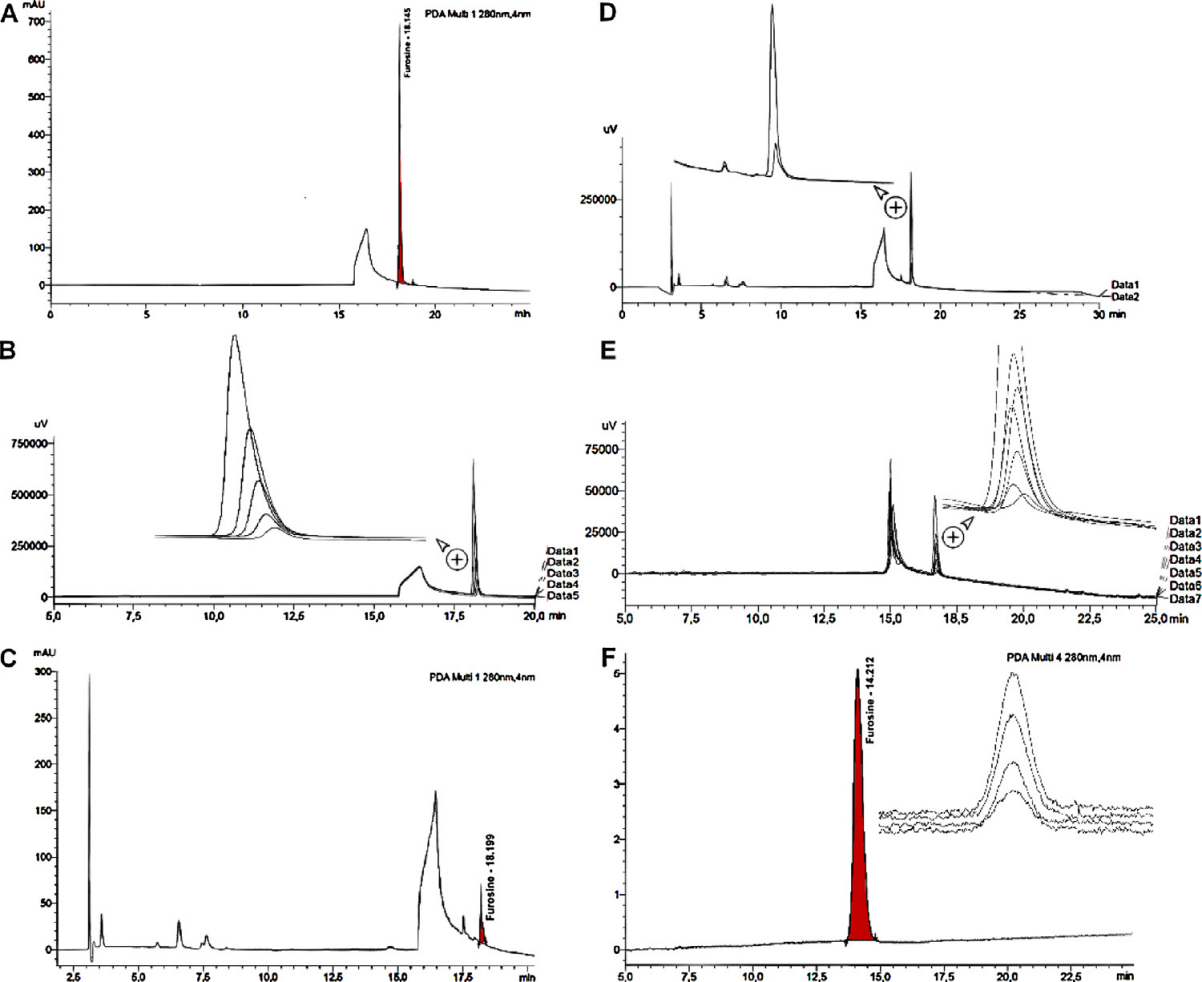
Furosine HPLC analysis with the proposed method of A. 100 mg L^−1^ spiked milk sample; furosine *R_t_* = 18.145 min B. Progressive concentration increase for a five-point calibration curve ranging from 5 to 100 mg L^−1^ C. Commercial milk sample (2 g/100 mL fat, UHT); furosine *R_t_* = 18.199 min sample interpolated concentration 3.02 mg L^−1^ D. Comparison between a commercial milk sample vs the same sample when spiked; an increase in the area for the signal at ca. (18.2 ± 0.1) minutes is evident. E. Extinction experiment to assess sensibility (i.e., furosine levels near calculated LoD) assaying concentrations of 10.28, 5.14, 4.11, 3.60, 2.06, 1.03 and 0.51 mg L^−1^. F. Furosine HPLC assay based on strong cation exchange column analysis, a 20 mg L^−1^ standard (*R_t_* = 14.212 min) and a 0.2 to 0.8 mg L^-1^extinction assay are shown.

Chromatographic separation was achieved using a 250 mm × 4.6 mm, 5 μm analytical column (SUPELCOSIL^™^, LC-8, 58297, SUPELCO, St. Louis, MO, USA) and a solvent system that included a 5 mmol TsOH or HSA L^−1^ solution (resulting in a 2.3–2.4 pH value, A) and ACN (B). Gradient elution was accomplished as follows: 100% A, 0–10 min; 50% A, 10–20 min; 50% A, 20–22 min and 100% A, 22–27 min. Solvent flow and column compartment temperature, detector wavelength and sample injection volume were kept constant during elution at 1.0 mL min^−1^, 30 °C, 280 nm, and 20 μL, respectively (Fig. 1A).

### Chromatographic conditions using a strong cation exchange column

A second and independent separation was successfully performed using a Zorbax 300-SCX 250 × 4.6 mm and 5 μm (Agilent Technologies, Santa Clara, CA, USA). A 0.2 mol L^−1^ sodium phosphate buffer at pH = 3.0 was used to achieve furosine detection at 280 nm with an injection volume of 5 μL (Fig. 1F), a flow rate of 1.0 mL min^−1^, and a column compartment set at 30 °C.

### Sample treatment and clean-up

Sample treatment was performed according to the ISO 18,329 IDF 193 reference method. Briefly, to a fluid milk subsample of 2 mL, in a 40 mL glass vial (27184 SUPELCO, St. Louis, MO, USA), 6 mL of an HCl 10.6 mol L^−1^ aqueous solution is added. Immediately thereafter, the vial is capped with a septum, tan PTFE/silicone (27188-U, SUPELCO, St. Louis, MO, USA) and nitrogen is bubbled for 1 min (to purge any oxygen), into the solution through a needle. The resulting mixture was heated adiabatically for 23 h at 110 degrees Celsius. The resulting hydrolysate was filtered through a qualitative filter paper, grade 4 (Whatman^®^, GE Healthcare Life Sciences Pittsburgh, PA, USA) by gravity. Subsequently, 0.5 mL of the hydrolysate was filtered through a SPE cartridge previously conditioned with 5 mL MeOH and 10 mL water (WAT020805, Sep-Pak VAC C_18_ 3 cc 500 mg, Waters Corporation, Milford, Massachusetts, USA).

Dropwise elution from the SPE cartridge was performed using a 3 mL HCl 3 mol L^−1^ solution. The eluate was pressure-filtered through a 0.20 μm filter (17764-Q, Minisart-RC25^®^ syringe filters with regenerated cellulose hydrophilic membrane, Sartorius AG, Göttingen, Germany) and recovered in an HPLC 2 mL vial for injection (Shimadzu Prominence). As recommended in the reference ISO 18,329 IDF 193 method, the remaining of the hydrochloric acid hydrolysate is used for protein determination according to method AOAC OMA^SM^ 991.20 Nitrogen (Total) in Milk. Kjeldahl Method.

Proper reconstitution has a profound effect on the performance parameters for the determination of furosine in milk powder. When 0.2 g of the solid sample is directly spiked with a previously prepared furosine and diluted with the 6 mol L^−1^ HCl solutions, poor accuracy (i.e., 15.3–46.7%) and reproducibility are obtained. However, when the same sample (0.2 g) is first suspended into a volume of 2 mL of water, using an Ultra-Turrax^®^ (IKA^®^ T10 basic, IKA Works, Inc, Wilmington, USA) at 8 500 rpm, before spiking and dilution with the HCl solution, the resulting recoveries improve drastically (i.e., 81.5–89.6%, Table 2). It is noteworthy that milk powder is estimated to be 10 times more concentrated in solids, including protein, than a liquid sample. Thus, then differences in the reconstitution directly affect the recoveries. Small sample quantities can be used when assaying powdered milk as it usually exhibits higher concentrations of furosine due to more severe heat treatment compared to fluid milk.

### Considerations for ion pair chromatography

Furosine contains two amine functional groups that can interact with a sulfonic acid group. The proton transfer from the acid to the amine groups provides a fast and essentially complete reaction with no structural rearrangements/changes for furosine and produces a stable ion-pair, which can be quantitatively analyzed due to the interaction that exists between the aromatic moiety of the sulfonate and the alkyl chain of the bonded phase of the column. Both the sulfonate and the sulfonate-furosin ions have been demonstrated to be inert toward the chromatographic column, and the reagent produces no byproducts during ion pairing, providing better chromatographic efficiency than that obtained from the acetate suggested by the reference method. Organic acids (e.g., trifluoroacetic acid) are commonly used during reverse phase chromatography to improve peak symmetry. We believe that in our HPLC method, the use of aromatic sulfonic acids such as TsOH serves two purposes: a) aromatic sulfonate anions exhibit different surfactant properties than other commonly used agents (e.g., heptane sulfonic acid) [18], b) the use of high purity reagents prevents common issues during ion-pair chromatography such as “ghosting” [18].

### Considerations for strong cation exchange chromatography

Polymer-bound sulfonic acids are found both commercially (e.g., 532312, Sigma-Aldrich) and as the stationary phase in several cation exchange columns (e.g., Zorbax 300-SCX). Considering the existence of the latter column, we postulated that it may well retain furosine by a mechanism similar to TsOH ion-pairing, but occuring *in situ* within the column; on the solid phase. In contrast with other methods, no organic solvent or ion pair agent is needed during the separation on a SCX column and, as such, this method is less expensive and might be considered as green chemistry. Another advantage resides in that the column uses isocratic mode during separation, this prevents baseline drift during phase shifting (i.e., a steadier baseline, compare Fig. 1 panels A through E vs. panel F). Interestingly, similar retention times are rendered for both methods (i.e., 18.2 ± 0.1 vs. 14.1 ± 0.1 min).

### Method optimization, validation and performance parameters

Five-point calibration curves were prepared each time measurements were performed. Concentrations ranged from 5.14 to 102.80 mg furosine L^−1^ (Fig. 1B). The general equation that resulted from three different calibration curves prepared independently under reproducibility conditions (i.e., on different days) is shown in Table 1. Mathematically, a limit of detection and quantification can be attained when considering the standard error of the calibration curve intercept divided by the slope, times 3.3 and 10, respectively (i.e., 0.59 and 1.79 mg L^-1^ for the TsOH method, Table 1). In turn, when considering the matrix and calculation to obtain the result expressed within a fluid milk sample the limit of detection then turns, for example, into 1.48 mg furosine per 100 mL sample (Table 1). The lower limit was corroborated experimentally in an extinction assay, and the determined values (i.e., 0.58 for TsOH and 0.22 for SCX method, Table 1 and Fig. 1E, F) are consistent with the previous calculations.

**Table 1:** Work range and linearity comparison for two pairing reagents for furosine.

*Parameter*	*Strong cation exchange*	*Pairing reagent*	
		*TsOH*	*HAS*
General equation (*n* = 3)	*y* = (1.2 × 10^4^ ± 1.5 × 10^2^)*x* − (1.6 × 10^3^ ± 2.1 × 10^2^)	*y* = (5.0 × 10^4^ ± 8.9 × 10^2^)*x* − (1.7 × 10^4^ ± 2.0 × 10^3^)	*y* = (4.8 × 10^4^ ± 6.6 × 10^2^)*x* + (3.5 × 10^4^ ± 1.3 × 10^3^)
Coefficient of determination (*r*^2^, Pearson)	0.9999	0.9997	0.9993
Theoretical plates (*N*)	88436	139509	246942
Tailing factor	1.11	1.35	1.29
S/N	74.61	102.8	301.3
aLoD, mg L^−1^	0.22 (0.56)	0.58 (1.48)	1.70 (4.30)
aLoQ, mg L^−1^	0.67 (1.70)	1.78 (4.49)	3.75 (9.47)

a Values obtained based on the regression analysis (i.e., resulting directly from the variability from the calibration curves, in mg L^−1^). Conversely, data in parenthesis represent limits expressed in the sample; mg furosine per 100 mL sample.

**Table 2:** Repeatability and accuracy for furosine detected in commercial milk samples using the proposed methods.

*Repeatability (TsOH)*		
Matrix (fat content^a^, number of independent samples tested)	Average, mg furosine/100 g milk protein	RSD, %
Milk powder (27.4% fat, *n* = 8)	455.3	2.3
Whole milk (3.2% fat, *n* = 1, 3 replicates)	133.8	4.1
Partially skimmed milk (1.9% fat, *n* = 4, 3 replicates each)	246.2	2.7
	261.8	3.8
	280.6	3.2
	310.0	2.8
Skimmed milk (0.3% fat, *n* = 1, 3 replicates)	390.9	1.3

*Accuracy*			
Matrix	Fortification level, mg furosine/100 g milk protein	Concentration, mg furosine/100 g milk protein (Recovery, %)^a^	
		*TsOH*	*SCX*
Milk powder (27.4% fat)	0.0	84.2	88.0
	12.3	86.0 (89.1)	89.8 (89.6)
	39.3	100.7 (81.5)	105.9 (83.1)
	106.9	160.0 (83.3)	168.0 (85.8)
Fluid milk (1.9% fat, Ultra High Temperature)	0.0	23.24	22.21
	6.9	30.1 (100.0)	29.6 (101.8)
	22.6	43.5 (92.6)	43.0 (95.9)
	64.0	82.6 (94.7)	86.2 (100.1)

*Robustness (Interlaboratory comparison)*			
	Concentration, mg furosine/100 g milk protein^b^ ± RSD, %		
Sample/Method	*MET-058*^d^	*TsOH*^c^	*SCX*^c^
Whole milk	265.4	196.3 ± 19.9	192.6 ± 6.6
	271.3	291.9 ± 6.6	265.4 ± 4.4
Partially skimmed milk	275.0	211.0 ± 13.2	241.9 ± 5.1
	189.7	174.3 ± 2.2	190.4 ± 2.2
	355.9	239.7 ± 11.0	302.9 ± 11.0
	218.4	161.8 ± 8.1	183.8 ± 2.2
	272.1	269.1 ± 8.1	322.8 ± 12.5
	272.8	197.1 ± 5.9	224.3 ± 2.2
	386.0	302.9 ± 3.7	297.8 ± 15.4
Skimmed milk	467.6	422.1 ± 25.0	443.4 ± 2.9
	491.9	358.8 ± 36.8	353.7 ± 14.7
	358.1	247.8 ± 2.9	288.2 ± 2.2

Intermediate precision (Average, mg furosine/100 g milk protein ± RSD, %)^e^		
Condition modified/method	*TsOH*	*SCX*
Chromatographic system	261.3 ± 1.6	285.2 ± 1.4
Column Batch	272.4 ± 1.2	265.2 ± 1.9
Analyst	259.1 ± 2.4	289.6 ± 0.9

a Fat values resulted from a year nation-wide survey with *n* = 19 samples for each matrix type, RSD < 10.4% and < 16.5% for fluidized milk and milk powder, respectively.

b Data is the result of three independent replicates, mean values are shown, RSD < 3.5% for all cases.

c All samples weighted for in-between method comparison.

d The same samples analyzed by the proposed methods were submitted to Muva-Kempten GmbH (Allgäu, Germany) laboratory for comparison. According to their analysis report, the laboratory uses an HPLC method (MUVA-MET058 Ital. law Gazette No. 162).

e Data obtained using the same sample (i.e., a partially skimmed milk), and the protocol described above, but under different conditions (all six tests performed on different days).

Moreover, an additional assay was performed using HSA sodium salt instead of TsOH to compare the proposed method with an already established approach (Table 1). We found that HSA prepared curves presented lower RSDs for the intercept and the slope (i.e., 3.7 and 1.4, respectively) when compared with those made with TsOH. (i.e., 11.8 and 1.8, respectively). Considering the magnitude of the slopes obtained, both reagent and column based linear regressions seem to be equal; linearity is sustained even at 200 mg L^−1^. However, regarding sensitivity TsOH is 2.9 (i.e., 1.70/0.58) fold acuter than its HSA counterpart and, in turn, SCX is 2.6 times more sensitive than the former (i.e., 0.58/0.22, Table 1). Furthermore, *p*-toluensulfonic acid monohydrate is almost half the expense of sodium 1-heptanesulfonate (e.g., 28.10 and 40.90 USD, respectively, for 5 g product, same quality reagent). In the case of the peak symmetry, the signal obtained during SCX has the least tailing factor of the three methods compared; possibly because it involves the direct interaction of the stationary phase with the analyte. Meanwhile, ion-pair separation relies on multiple interactions occurring at once (i.e., ion-pair/analyte and ion-pair/column stationary phase).

Repeatability was attained for four types of milk samples, (i.e., milk powder, whole milk, partially skimmed milk and skim milk) values for RSD obtained ranged from 1.31 to 4.06%. No significant differences in variability among milk samples (Mann-Whitney *U*, *p* < 0.05, Table 2) were found, and all values are below the reference method maximum threshold for repeatability (i.e., 6.02 expressed also as RSD). When calculating reproducibility for methods destined for furosine determination, change in values during storage time, even under preservation should be considered. Furthermore, values locally obtained for furosine in mg per 100 g milk protein (Table 2), seem to be in line with those reported in the literature [19].

Method accuracy was determined as the recovery of three different concentration levels for furosine (i.e., ca. 2, 5, and 15 mg L^−1^ and 1, 3, and 9 mg L^−1^ for milk powder and fluid milk, respectively) (Table 2, Fig. 1D). Recoveries fluctuated from 81.5 to 100.0% and 83.1 to 101.8% for TsOH and SCX methods, respectively. Said values are considered adequate for the concentrations assayed according to US FDA [20] and ICH [21] validation criteria. Data obtained herein are in line with those obtained from the same samples analyzed by another laboratory (see Table 2 footnote *d*), as Pearson test show variables with a positive association (*r* = 0.891; *p* < 0.001 which indicate that both variables tend to increase together, see robustness Table 2). No significant differences were found when a same group of samples were tested using MET-058, TsOH or SCX (*p* > 0,05) However, slight variations among laboratories may be caused by i. methodological differences, ii. storage temperature during transport (i.e., samples assayed in Costa Rica versus those transported and analyzed in Europe, strict abidance to or differences in cold chain/storage temperatures [22]) and iii. dates between assays.

These methods aim to establish an accurate and reliable technique which will allow assessing basal furosine concentrations in commercial milk (Fig. 1C) over the country. Later on, the same procedures will be used to distinguish among storage related levels vs. typical thermal treatment vs. adulteration of fluid milk with reconstituted milk powder [23,24]. However, in this particular case method validation reliance on spiking limits and hinders robustness assaying since no commercial reference materials or proficiency tests are available for furosine (Fig. 1D).

It is important to notice that furosine alone may not be sufficient to achieve such goal. It is true that multiple or prolonged thermic treatment will increase furosine concentrations. Still, to our knowledge, no legislation has been established. For example, some countries even permit the commercial preparation and sale of recombined milk (e.g., Nicaragua [25]), but it must be labeled as such. In contrast, in Costa Rica adding milk powder to fluid milk is considered adulteration. Due to their structural relatedness, the proposed methods may be very well suited for other measurements linked to Maillard reaction (e.g., homoarginine [reactive lysine], *N*^6^-carboxymethyllysine) [2]. Noteworthy, our data seems to hint a possible role in the milk skimming on the amount of furosine encountered. As the fat is removed, the more protein thermal susceptibility is observed (i.e., furosine in whole milk < partially skimmed milk < skimmed milk, Table 2). Finally, considering costs, ease of application, number of involved steps, high sensibility (Table 2) and the cleanness (no baseline drift nor interferences present) of the resulting chromatograms (Fig. 1F), the direct SCX approach is recommended.

### Statistical analysis

Calibration curves parameters (i.e., slopes and intercepts), coefficients of determination, limits of detection, and standard errors were computed as a linear fit model using SAS JMP 13 (Marlow, Buckinghamshire, England). A Mann-Whitney test was used to evaluate the hypothesis that variability among milk samples was equal. A One-way ANOVA and a Tukey post hoc test were used to compare differences among method results for a group of samples tested (the same samples were tested using 3 different methods as a way to assess robustness). Additionally, a Pearson product-moment correlation test was used to compare results obtained from a third-party method (MET-058) and our data (TsOH and SCX). For all tests, significant results were considered if *p* < 0.05.

### Calculations

Furosine is calculated as follows:

$$\left(\frac{Interpolated\;furosine,\;\frac{mg}{L}∙diluton\;factor\;(i.e.,\;\frac{3\;mL\;}{0.5.mL})}{cartridge\;recovery\;factor\;(e.g.,\;0.95)}\;∙8\;mL∙\frac{1\;L}{1\;000\;mL}/2\;mL.(milk\;aliquot)\right)∙100=mg\;furosine/100\;mL\;sample$$

If the result is to be reported in mass (i.e., mg furosine⁄100 g sample), milk density must be accounted for.

To express as a protein base:

$$\left(\frac{Interpolated\;furosine,\;\frac{mg}{L}∙diluton\;factor\;(i.e.,\;\frac{3.mL.}{0.5.mL})}{cartridge\;recovery\;factor\;(e.g.,\;0.95)}\;∙8\;mL∙\frac{1\;L}{1\;000\;mL}/4∙protein\;content\;of\;2\;mL.hydrolysate\;(in\;grams)\right)∙100=mg\;furosine/\;100g\;milk\;protein$$

Protein is determined as directed by AOAC 991.20 using 6.38 as a nitrogen-to-protein conversion factor.

## References

[1] Erbersdobler H.F., Somoza V. (2007). Forty years of furosine – forty years of using Maillard reaction products as indicators of the nutritional quality of foods. Mol. Nutr. Food Res..

[2] Mehta B.M., Deeth H.C. (2016). Blocked lysine in dairy products: formation, occurrence, analysis, and nutritional implications. Compr. Rev. Food Sci. Food Saf..

[3] Schmidt A., Boitz L.I., Mayer H.K. (2017). A new UHPLC method for the quantitation of furosine as heat load indicator in commercial liquid milk. J. Food Compos. Anal..

[4] Tirelli A. (1998). Improved method for the determination of furosine in food by capillary electrophoresis. J. Food Prot..

[5] Kulmyrzaev A., Dufour É. (2002). Determination of lactulose and furosine in milk using front-face fluorescence spectroscopy. Lait.

[6] Troise A.D., Fiore A., Wiltafsky M., Fogliano V. (2015). Quantification of Nε-(2-furoylmethyl)L-lysine (furosine), Nε-(carboxymethyl)-L-lysine (CML), Nε-(carboxyethyl)-l-lysine (CEL) and total lysine through stable isotope dilution assay and tandem mass spectrometry. Food Chem..

[7] Chiang G.H. (1988). High-performance liquid chromatographic determination of ε-pyrrole-lysine in processed food. J. Agric. Food Chem..

[8] [ISO] International Standardization Organization (2004). Milk and Milk Products - Determination of Furosine Content - Ion-Pair Reverse-Phase High-Performance Liquid Chromatography Method.

[9] Resmini P., Pellegrino L., Battelli G. (1990). Accurate quantification of furosine in milk and dairy products by a direct HPLC method. Ital. J. Food Sci..

[10] Resmini P., Pellgrino L. (1994). HPLC of furosine in for evaluating Maillard reaction damage in skimmilk powders during processing and storage. Bull. Int. Dairy Fed..

[11] Resmini P., Pellegrino L., Cattaneo S. (2003). Furosine and other heat treatment indicators for detecting fraud in milk and milk products. Ital. J. Food Sci..

[12] Delgado T., Corzo N., Santa-María G., Jimeno M.L., Olano A. (1992). Determination of furosine in milk samples by ion-pair reversed phase liquid chromatography. Chromatographia.

[13] Boitz L.I., Mayer H.K. (2015). Evaluation of furosine, lactulose and acid-soluble β-lactoglobulin as time temperature integrators for whipping cream samples at retail in Austria. Int. Dairy. J..

[14] Boitz L.I., Mayer H.K. (2016). Analytical assessment of the intense heat load of whipping cream, coffee cream, and condensed milk at retail in Austria and Germany. Dairy Sci. Technol..

[15] Erbersdobler H.F., Zucker H. (1966). Untersuchungen zum Gehalt an Lysin und verfugbarem Lysin in Trockenmagermilch. Milchwiss.

[16] Boucher S.E., Pedersen C., Stein H.H., Schwab C.G. (2008). Evaluation of the furosine and homoarginine methods for determining reactive lysine in rumen-undegraded protein. J. Dairy Sci..

[17] Mayer H.K., Raba B., Meier J., Schmid A. (2010). RP-HPLC analysis of furosine and acid-soluble β-lactoglobulin to assess the heat load of extended shelf life milk samples in Austria. Dairy Sci. Technol..

[18] Cecchi T. (2008). Ion pairing chromatography. Crit. Rev. Anal. Chem..

[19] Van Renterghem R., De Block J. (1996). Fursine in consumption milk and milk powders. Int. Dairy J..

[20] US FDA (2015). Analytical Procedures and Methods Validation for Drugs and Biologics, Guidance for Industry. https://www.fda.gov/downloads/drugs/guidances/ucm386366.pdf.

[21] ICH, Harmonized Tripartite Guideline/Guidance for Industry (2018). Q2(R1) Validation of Analytical Procedures: Text and Methodology.

[22] Pellegrino L., De Noni I., Resmini P. (1995). Coupling of lactulose and furosine índices for quality evaluation of sterilized milk. Int. Dairy J..

[23] Das S., Goswami B., Biswas K. (2016). Milk adulteration and detection: a review. Sens. Lett..

[24] Singuluri H., Sukumaran M.K. (2014). Milk adulteration in Hyderabad, India – a comparative study on the levels of different adulterants present in milk. J. Chromatogr. Separat. Techniq..

[25] Comisión Nacional de Normalización Técnica y Calidad, Ministerio de Fomento, Industria y Comercio (2011). Norma Técnica Obligatoria Nicaraguense. Leche Pasteurizada. http://legislacion.asamblea.gob.ni/normaweb.nsf/($All)/874E61383812793506257D8200724DA?OpenDocument.

